# Supplementary material for: Direct encapsulation of biomolecules in semi-permeable microcapsules produced with double-emulsions
Source: Sci Rep. 2022 Dec 10;12:21391. doi: 10.1038/s41598-022-25895-8 (PMC9736714; doi:10.1038/s41598-022-25895-8)
Supplement: Supplementary file 1 — Supplementary Information. [file 41598_2022_25895_MOESM1_ESM.pdf]

Direct encapsulation of biomolecules in semi-permeable microcapsules produced with double-emulsions

Grégoire Michielin and Sebastian J. Maerkl*

Institute of Bioengineering

School of Engineering

École Polytechnique Fédérale de Lausanne

E-mail: sebastian.maerkl@epfl.ch

Supporting Information

Materials and Methods

Supplementary Figures and Tables

Materials and methods

Microfluidic chip fabrication

The PDMS microfluidic devices were produced using standard soft lithography methods based on designs previously published[1,2]. Transparency films obtained from the SMaL lab from Prof. Esther Amstad at EPFL were used as photo masks for the production of a SU-8 master on a silicon wafer. A master was prepared using photolithography with the negative photoresist SU-8. A first 40 μm layer of 3025 SU-8 (Microchem) containing the inner and middle phase channel was spin-coated and UV exposed on the first half of the master while the second half was fully exposed. A second 60 μm layer of 3050 SU-8 (Microchem) was spin-coated and UV exposed aligned on top of the features to generate the main channels on both halves of the master. After using the master for PDMS replica moulding, both halves of the device were treated with oxygen plasma for 45 s and bonded to one another.

Microfluidic capsules production

The microfluidic system was operated using syringe pumps (New Era) controlled via a python script written in Professor Adam Abate's lab at UCSF. Syringes were connected to Tygon tubing and the tubing was connected to the chip via metal pins. The flowrates for the inner, middle and outer phase were set to 250, 200, and 2500 μL/h. The inner phase and outer phase are composed of a 10 wt/wt% aqueous solution of PVA (poly(vinyl alcohol), 13000-23000 g/mol, Sigma-Aldrich). Droplet production was observed at the junction and at the collecting channel through a 4x objective with a high-speed digital camera (Fastec HiSpec). The resulting double-emulsions were placed on a cell-counting slide and inspected with a 20x objective in brightfield and fluorescence. The double-emulsions were collected in UV-transparent cuvettes (UVettes, Eppendorf) and illuminated for 30 seconds from the side with the complete spectrum of a 100W Mercury lamp focused on the cuvette. Alternatively, an Omnicure S1500 200W UV curing lamp with standard filter (320nm-500nm) was used

and the double-emulsions were illuminated with a probe placed on the top of the cuvette. The polymerized capsules were transferred to a 1.5mL Eppendorf tube and washed 10 times in 1 mL cold wash buffer (1xPBS, 0.1 % Tween20) by centrifugation at 2000 rpm for 1.5 minutes at 4°C. Capsules were stored at 4°C in wash buffer.

Semi-permeable capsules

For the production of semi-permeable capsules, the middle phase was supplemented with 15 % porogen butyl acetate or 1-decanol (both from Sigma-Aldrich), 1 % Span80 (Fluka) surfactant and 4 % 2-Hydroxy-2-methylpropiophenone (97%, Sigma-Aldrich) photoinitiator. In the direct encapsulation of fluorophores, we supplemented the 10 % PVA inner aqueous phase with 100 μg/mL of 500 kDa FITC-dextran and 50 μg/mL TMR-dextran.

Fluorophore-containing capsules were washed as previously described and kept in wash buffer at 4°C overnight. The capsules were pipetted onto a cell-counting slide (Countess, Thermofisher) and fluorescence images were acquired in the Cy3 and FITC channels. For permeability characterization, empty capsules were immersed in a solution containing 10 kDa RITC-dextran and 32.7 kDa EGFP. After different incubation times, fluorescence images were acquired in the Cy3 and FITC channels.

Direct encapsulation of proteins and enzymes

For direct encapsulation of proteins, the 10 % PVA inner phase was supplemented with 32.7 kDa recombinant EGFP (Biovision) mixed to a final concentration of 2 μg/mL or 60 kDa FITC-labeled streptavidin (Biolegend) to a final concentration of 50 μg/mL. Double-emulsions or polymerized capsules were loaded in a cell-counting chamber and imaged on a microscope with 20x magnification.

For direct encapsulation of enzymes, econoLuciferase 10 mg/mL solution (Biosynth) was mixed to 4 v/v % in the 10 % PVA inner phase. The double-emulsions and polymerized capsules were loaded in a cell-counting chamber and inspected on a microscope and fluo-

resence signal was measured. In the bioluminescent assay, 25 μL of blank, empty capsules, econoLuciferase-containing capsules, or free econoLuciferase were mixed with 100 μL of luciferase assay reagent (Promega) in a black 96-well plate and imaged for 50 minutes on a plate reader. The bioluminescent signal was integrated over the duration of the experiment. β-galactosidase aqueous glycerol suspension (Sigma-Aldrich) was mixed to 10 v/v % in the 10 % PVA inner phase. The produced capsules were resuspended in a 0.5 M trehalose solution and 15 μL drops were pipetted onto a silicon mat and dried overnight in a 37°C incubator. The resulting dried pellets were placed in a tube and rehydrated with a chlorophenol red β-D-galactopyranoside (CPRG) solution. The color change was imaged with a mobile phone camera, or with a color camera (Pike) mounted on a microscope and imaged with a 4x objective.

DNA-strand displacement reactions

We used capsules containing 60 kDa FITC-labeled streptavidin for the immobilization of biotinylated DNA strands. The DNA strands were synthesized by IDT with HPLC purification. The DNA sequence and DNA modifications were strictly identical to the ones presented in Joesaar *et al.*[3] and we followed the same protocol for the assembly of two populations of capsules containing the first (F_1) and second (F_2) gate DSD reactions. Briefly, 40 μl of a dispersion of FITC-streptavidin containing capsules, 20 μl of 4x buffer and 8 μl of biotinylated DNA gate strand (F_1 or F_2, from a 10 μM stock solution) were mixed with a pipette in a 1.5 ml Eppendorf tube and incubated at room temperature for 1 h, followed by addition of 12 μl output strand (Q_1 or Q_2, from a 10 μM stock solution), gentle mixing and overnight incubation at 4°C. The excess unbound output strand was removed by removing around 50 μl of the supernatant and the capsules were washed 3 times with 400 μl of buffer by centrifugation at 1500 rpm, 4°C, and the capsules with the immobilized DSD reaction were stored at 4°C. To perform the double-layer signaling cascade, the two capsules populations were mixed and supplemented with a fuel strand. After the addition of an input strand (A)

at a concentration of 50 nM, 10 nM or 100 nM, the resulting solution was pipetted in a cell counting chamber (Countess, Thermofisher). To prevent evaporation, the device was placed in a petri dish and surrounded by kimwipes saturated with 1xPBS, allowing for long-term imaging of the two-layer signalling cascade. The fluorescent signal for the first gate (Cy5) and second gate (Cy3) was measured with a 200 ms exposure every 30s for a duration of 1 hour. The resulting images were captured in a 3x3 stitch with a 20x objective. For an estimation of the signal increase over the timecourse of the experiment, we used ImageJ and applied the "Find edges" process followed by thresholding using the default settings to detect the capsules in each image. We used erosion three times followed by dilation three times to remove smaller detected particles. Then, the process "fill holes" was used once, followed by 5 x erosion, 3 x dilation, 5x erosion, 3 x dilation, 5 x erosion. We finally used the "analyze particles" function to extract the median for particles with circularity over 0.5. For each timepoint of the experiment, the median intensity of every detected particle and the median intensity of all particles was plotted using PRISM.

Supplementary Figures and Tables

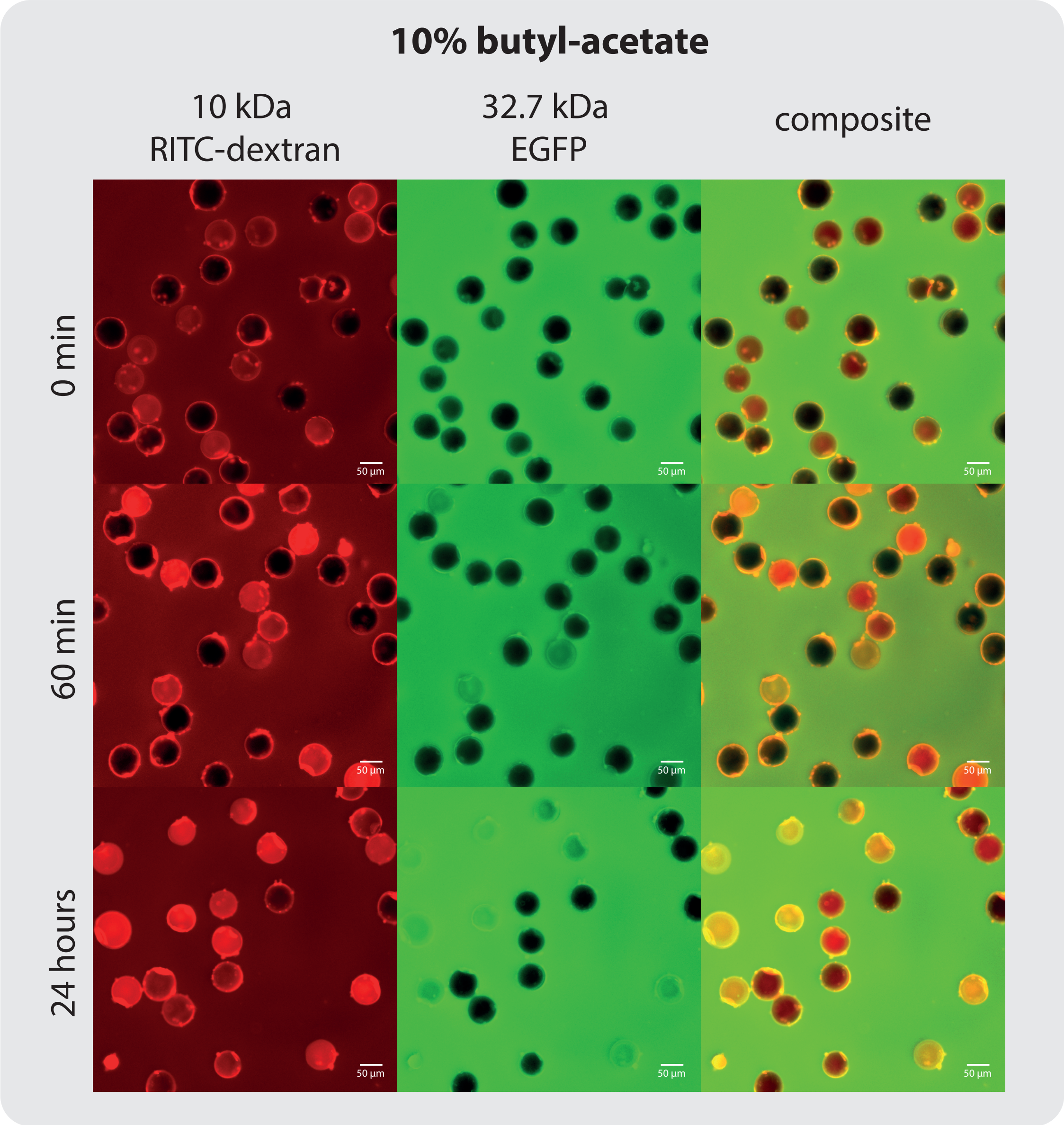

Figure S1: **PEG-DA 258 microcapsules produced with 10% butyl-acetate porogen.** The produced capsules were placed in a solution containing 10 kDa RITC-dextran and 32.7 kDa EGFP and imaged after 0 minutes, 1 hour, and 24 hours. Compared to the use of 15% butyl-acetate, the capsules displayed reduced permeability to 10 kDa RITC-dextran with a significant proportion of capsules without corresponding fluorescent signal after 1 hour of incubation.

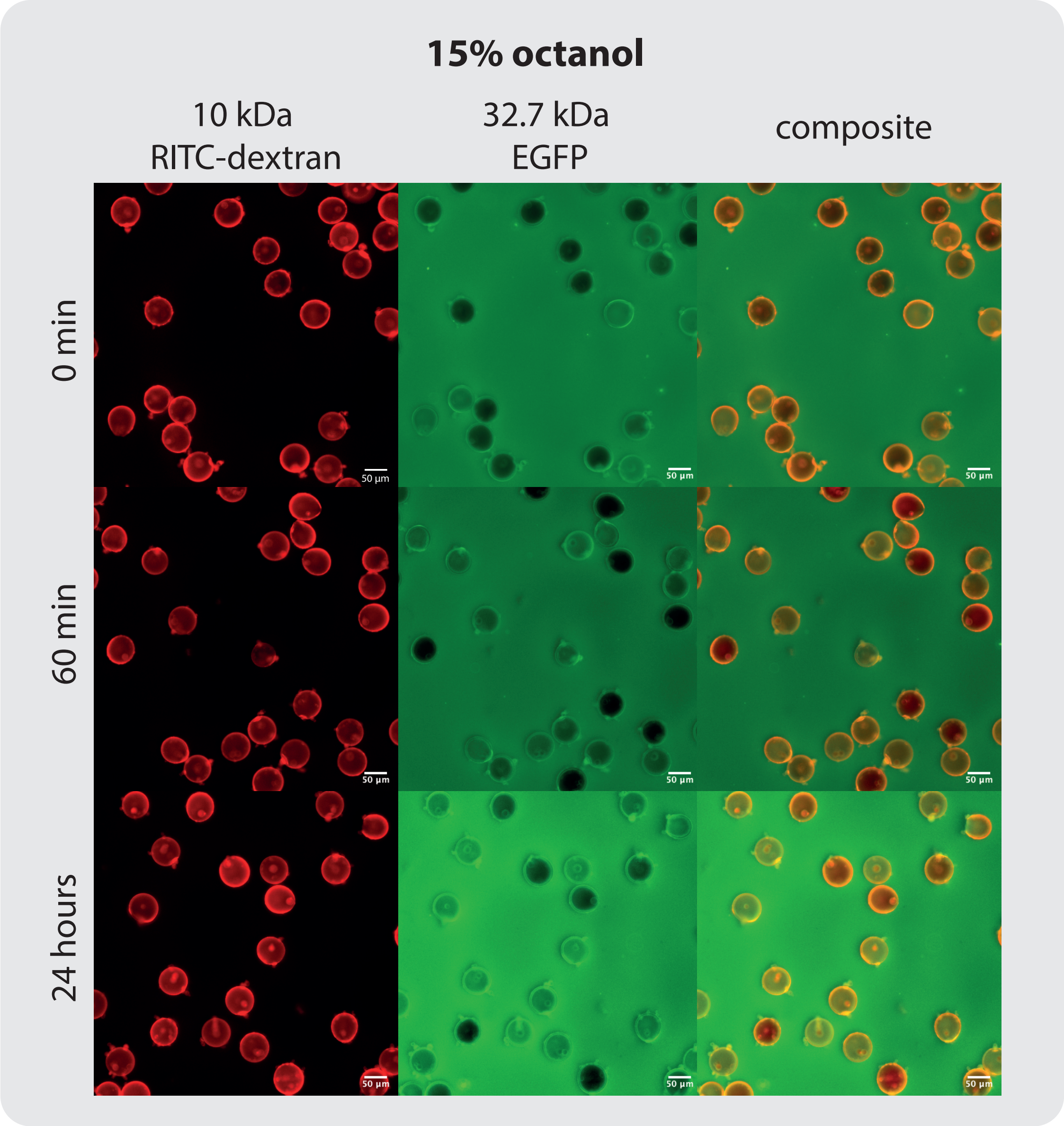

Figure S2: **PEG-DA 258 microcapsules produced with 15% octanol porogen.** The produced capsules were placed in a solution containing 10 kDa RITC-dextran and 32.7 kDa EGFP and imaged after 0 minutes, 1 hour, and 24 hours. Most capsules produced using 15% octanol show fluorescent signal corresponding to both 10 kDa RITC-dextran and 32.7 kDa EGFP after 24h of incubation.

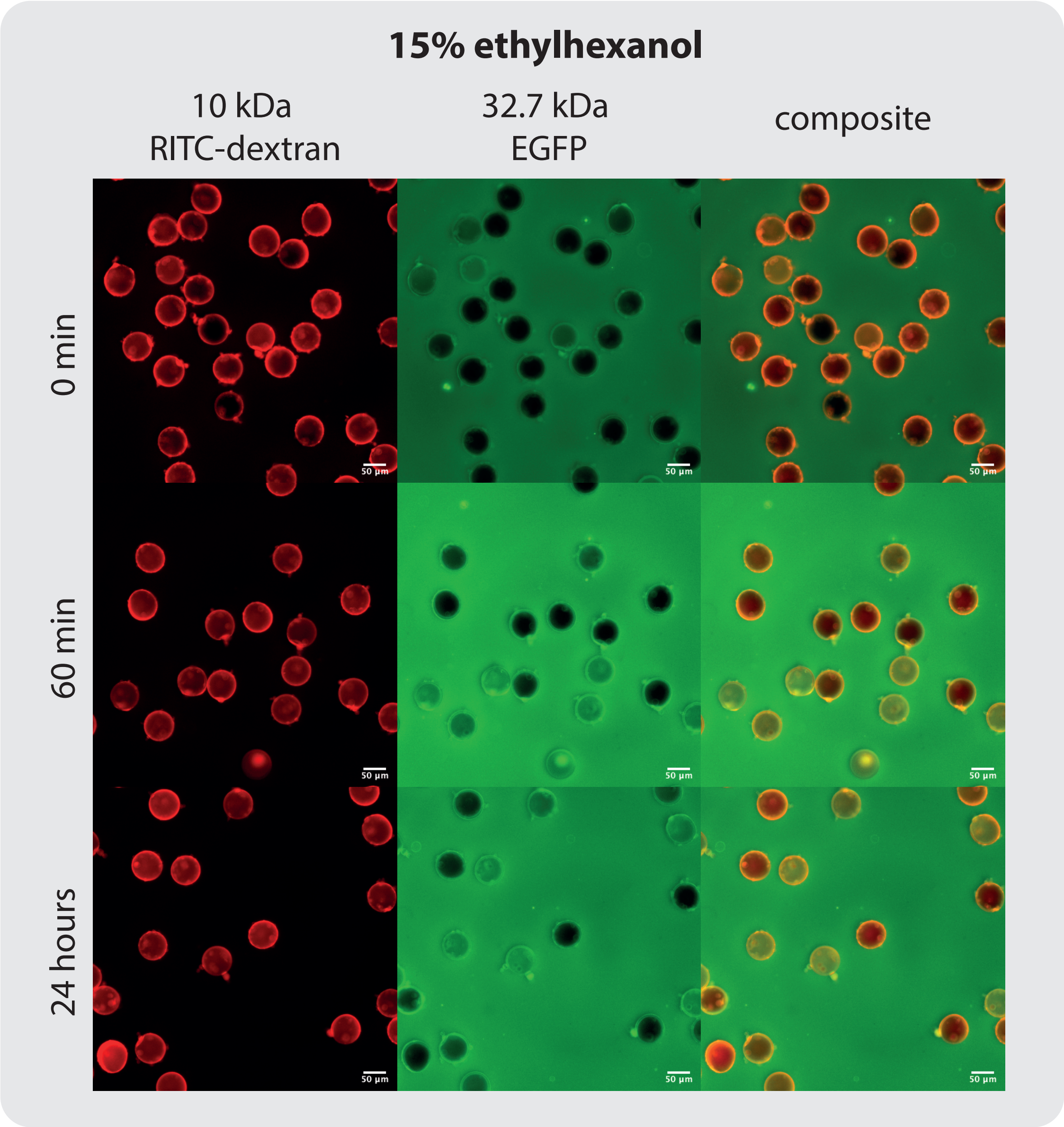

Figure S3: **PEG-DA 258 microcapsules produced with 15% 2-ethyl-1-hexanol porogen.** The produced capsules were placed in a solution containing 10 kDa RITC-dextran and 32.7 kDa EGFP and imaged after 0 minutes, 1 hour, and 24 hours. The capsules produced using 15% 2-ethyl-1-hexanol showed a similar proportion of capsules with fluorescent signal as capsules produced with 15% butyl-acetate.

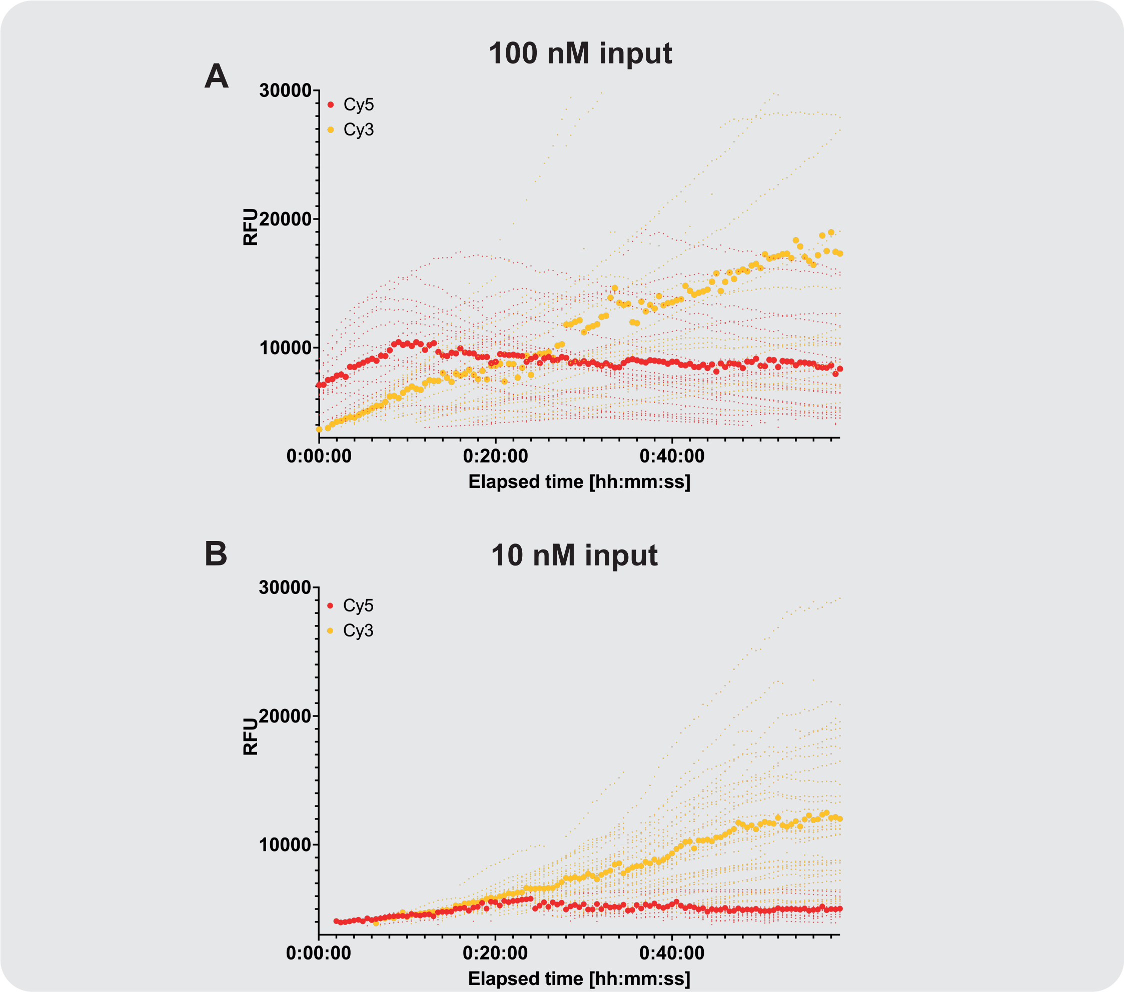

Figure S4: **Immobilization of DNA strand displacement reaction in semi-permeable microcapsules and implementation of a two-layer signalling cascade. (A)** Median intensity of detected particles following activation with 100 nM input. **(C)** Median intensity of detected particles following activation with 100 nM input. In both plots, the larger symbols correspond to the median of all detected particles in a given fluorescent channel.

References

(1) Assaf Rotem, Adam R Abate, Andrew S Utada, Volkert Van Steijn, and David A Weitz. Drop formation in non-planar microfluidic devices. *Lab on a Chip*, 12(21):4263–6, 2012.

(2) L R Arriaga, E Amstad, and D A Weitz. Scalable single-step microfluidic production of single-core double emulsions with ultra-thin shells. *Lab on a Chip*, 15(16):3335–3340, July 2015.

(3) Alex Joesaar, Shuo Yang, Bas Bögels, Ardjan van der Linden, Pascal Pieters, B V V S Pavan Kumar, Neil Dalchau, Andrew Phillips, Stephen Mann, and Tom F A de Greef. DNA-based communication in populations of synthetic protocells. *Nature Nanotechnology*, 14(4):369 – 378, 02 2019.
